# Long-term care insurance within married couples: Can’t insure one without the other?

**DOI:** 10.1007/s11150-025-09779-0

**Published:** 2025-05-23

**Authors:** Norma B. Coe, R. Tamara Konetzka, Chuxuan Sun, Courtney Harold Van Houtven

**Affiliations:** 1 Department of Medical Ethics and Health Policy, Perelman School of Medicine, University of Pennsylvania, Philadelphia, PA USA; 2 National Bureau of Economic Research, Cambridge, MA USA; 3 Department of Public Health Sciences, University of Chicago, Chicago, IL USA; 4 Department of General Internal Medicine, Perelman School of Medicine, University of Pennsylvania, Philadelphia, PA USA; 5 Department of Medicine, Duke University Medical Center, Durham, NC USA

**Keywords:** private long-term care insurance, purchase decisions, marriage, family care, long-term care risk, adverse selection, D1, D14, G52

## Abstract

Although long-term care remains one of the largest uninsured risks facing older Americans, demand for insurance remains low. While there is a long literature estimating a variety of factors that contribute to this low demand, much of it has overlooked the fact that most private long-term care insurance (LTCI) purchases are made within couples, adding a host of additional reasons for low demand. This paper examines the role of financial decision-making power within the couple and the association with LTCI purchase decisions. We document LTCI purchase patterns among married couples and find that, among couples who ever purchase LTCI, they are roughly equally likely to purchase for the woman exclusively (10.0%), the man exclusively (11%), or both (11%). However, among couples where women have more bargaining power, LTCI purchases are more likely overall (40% vs. 33%), and more likely to cover the woman, either exclusively (16% vs. 11%) or as part of both members of the couple (14% vs. 11%), than among couples with more traditional gender roles. In adjusted analyses, we find that women are more likely to be insured when they have more bargaining power. These findings suggest that intra-household bargaining power may be another potential explanation for the particularly low LTCI take-up, especially in the time period in which policies were unisex-priced.

## Introduction

The aging of populations worldwide has increased policy focus on the need for long-term care (LTC) and a sustainable way to finance it. Several high- and middle-income countries have adopted public LTC insurance (LTCI) regimes (Chen & Xu, 2020, Eggers & Xu, 2024, Geraedts et al., 2000, Chen et al., 2020, Yamada & Arai, 2020) or are piloting LTCI programs (Chen et al., 2021, Chen et al., 2023). In the US, while Medicaid currently covers half of total LTC expenditures (Reaves & Musumeci, 2015), LTC costs still constitute the largest financial risk facing older Americans today. Policymakers have long been interested in expanding private LTCI markets, yet currently only 14% of older Americans have private LTCI (KFF Polling, 2023). Washington State began the first public long-term care insurance program in the US in 2023, which has been met with considerable political opposition (Aaron, 2022, Washington State House Republicans, 2024, Warshawsky, 2022). With no clear policy solution in sight, it is important to understand consumer behavior in private LTCI markets.

There is a long literature exploring reasons for purchase or non-purchase of LTCI (Brown et al., 2012, Lockwood, 2018, Davidoff, 2010, Mellor, 2001, Mommaerts, 2024, Brown & Finkelstein, 2008, Finkelstein & McGarry, 2006, Ameriks et al., 2016, Ko, 2022, Braun et al., 2019). These papers explore a host of potential barriers to comprehensive coverage, including factors that decrease the supply and demand for insurance, such as cheaper or more flexible options than formal insurance policies. Although these papers have identified multiple separate factors that explain purchase behaviors, the combination of these factors does not fully explain the lack of private LTCI coverage. Thus, the LTCI puzzle remains.

In the broader economic context, there is substantial evidence that who has decision-making power within a couple influences the purchasing and consumption patterns of the household (Baland & Ziparo, 2017, Ambler et al., 2021). In cooperative household models, each individual receives a relative weight, or distributional factor, which influences the allocation of resources (Grossbard, 1984, Doss, 2013, Bourguignon et al., 2009, Himmelweit et al., 2013, Browning et al., 2014). Some empirical measures used to proxy these distributional factors include relative ages, relative education, individual incomes, social norms, traditional roles, and institutional variables, such as legal and welfare rules affecting the cost of marriage breakdown (Maitra & Ray, 2006, Bourguignon et al., 2009, Himmelweit et al., 2013, Bertocchi et al., 2014, Browning et al., 2014), while only a few papers measure distributional factors directly through surveys (Schmeer, 2005, Booysen & Guvuriro, 2021). In numerous countries, improvements in women’s economic bargaining power has led to greater expenditure on social goods such as food (Schmeer, 2005, Gummerson & Schneider, 2013, Opata et al., 2020 and education (Quisumbing & Maluccio, 2003, Basu & Maitra, 2020, Booysen & Guvuriro, 2021), and lower expenditure on vice-goods such as alcohol and cigarettes (Gummerson & Schneider, 2013, Menon et al., 2018, Opata et al., 2020, Basu & Maitra, 2020).

We do not yet know the role of intra-family bargaining in LTCI decisions. The majority of empirical work has focused on individual-level barriers to purchase, despite the evidence that long-term care insurance purchase decisions are usually made when individuals are married or coupled. For example, the average age of purchase is 59 and has been declining over time (AALTCI, 2023), ages at which most individuals are a part of a married or partnered couple. Qualitative work suggests that couples consider LTC planning and purchases of LTCI together (Stum, 2008, Sperber et al., 2014), and economic theory considers LTCI purchase in the family context (Pauly, 1990b, Zweifel & Struewe, 1998). The empirical literature to date has not.

Beyond marital status per se, which is positively associated with individual purchase patterns in the non-group market (McCall et al., 1998, Schaber & Stum, 2007), we know little about how being part of a couple influences LTCI purchase. Three studies find that having a working spouse is correlated with a lower likelihood of purchasing LTCI (Van Houtven et al., 2015, McCall et al., 1998, Coe et al., 2015). This finding is consistent with prior work that found that the ability to engage in risk-sharing within couples serves as a partial substitute for formal insurance markets, thereby reducing the value of private insurance (Kotlikoff & Spivak, 1981, Brown & Finkelstein, 2008).

We contribute to the stream of literature on LTCI decisions among older adults by describing purchase patterns of LTCI for couples and examining how female bargaining power is associated with the observed purchase patterns, using unique survey data identifying the financial decision-maker about insurance. First, we develop a conceptual model discussing why female bargaining power within a couple could produce different purchase patterns. Second, we examine empirically whether female bargaining power is correlated with LTCI, controlling for the demand- and supply-side factors that have been shown to be important in the literature. We find that dynamics within the couple are also important. Indeed, among couples who insure only one member of the family, the woman being the financial decision maker is the second largest positive predictor of insuring the woman, either exclusively or with the husband.

## Background and conceptual model

While LTC remains one of the largest uninsured risks facing older adults, there are public LTCI programs in the US. Individuals with a service-level disability may be eligible for LTCI through the Veteran’s Administration. Medicaid, the health insurance program for poorer individuals, covers unlimited nursing home use and an expanding number of home and community-based services (HCBS), although there is substantial variation among states in the eligibility, generosity, quality, and availability of LTC within the Medicaid program. Individuals must pass financial tests to be covered by Medicaid, and these financial limits depend on household size, marital status, and state of residence.

Private LTCI, the focus of this paper, covers costs associated with disability that may develop many years into the future; yet, due to health screening policies designed to minimize adverse selection, a LTCI policy must be purchased when the individual is healthy. LTCI typically covers nursing homes, and over time, coverage for additional services has become common, including home health care and assisted living facilities. LTCI products and pricing are regulated at the state level. While the vast majority of LTCI policies sold are individual policies, couples have the option of buying joint policies with lower premiums, often sharing the insured value. Traditionally, states barred insurers from differential pricing by sex, but starting in April of 2013, LTCI companies in most states were allowed to introduce sex-specific pricing (sex-neutral pricing remained in MT and CO).

### Empirical evidence on individual-level determinants of LTCI demand

One must be eligible to purchase a policy, and poor health is therefore a barrier to purchasing LTCI (Kim, 2009). Cornell et al. (2016) estimate that approximately 40% of the US population aged 50–70 could not qualify for a private LTCI policy. They also have to know about their risks, both financial and health. Brown et al. (2012) find that people vastly underestimate the probability of needing LTC in the future and the price of that care, and overestimate the insurance coverage provided through Medicare. Corroborating evidence was found in Coe et al. (2015), who find that individuals who have provided LTC to their parents and therefore become aware of the limits of Medicare coverage are more likely to buy LTCI for themselves.

Financial considerations, both current and future, could influence LTCI demand. LTCI is considered fairly expensive relative to the incomes of the majority of older adults. The average cost of a typical $165,000 benefit policy for a couple where both individuals are 60 at the time of purchase ranged from $2,550-$5,670 per year in 2023, depending on the escalation factor (AALTCI), while the median income of couples aged 65 and older was almost $85,000 in the same year (Census, 2024). Lockwood (2018) highlights how bequest motives, if they are considered luxury goods, increases the savings of richer retirees relative to poorer ones, and encourages self-insurance for these late-life risks, decreasing the demand for annuities and private LTCI. Davidoff (2010) posits that home equity may be a substitute for LTCI, especially when individuals are reluctant to move and borrowing against home equity at older ages is difficult.

Both theoretical (Pauly, 1990a) and empirical work (Mellor, 2001, Mommaerts, 2024) have demonstrated that having family as potential caregivers could lower the demand for LTCI. Research shows that daughters are more likely to provide care to mothers (Grigoryeva, 2014), and that care to step-parents and divorced fathers is relatively unusual (Pezzin & Schone, 1999). Since extra-household sources of family care are a substitute for formal LTC (Van Houtven & Norton, 2004, Charles & Sevak, 2005), LTCI demand should be higher with fewer potential extra-household family care providers.

Brown & Finkelstein (2008) show that Medicaid, by providing back-stop insurance, crowds out demand for private LTCI for a large swath of the wealth distribution. Brown & Finkelstein (2007a) calculate the money’s worth, or the value of LTCI, to determine if the pricing of policies was driving low demand. Using data from 2002, they found that the unisex pricing regulations meant that loads were much higher for men than for women – essentially, women were getting a discount. However, this did not lead to differences in coverage. The elimination of unisex pricing would decrease the subsidy to women and could further depress demand.

Research has also focused on the supply-side of the market and how it contributes to the lack of comprehensive coverage (Finkelstein & McGarry, 2006; Ameriks et al., 2016; Ko, 2022; Braun et al., 2019*)*. Some research has focused on the incompleteness of the product, for example, only covering a limited amount of costs or years of coverage, or other market imperfections as supply-side reasons for the low LTCI coverage. While these factors have been shown to lower demand, none account for the coverage to be as low as observed.

### Conceptual model

Pauly (1990a) developed the classic individual-level decision model of the LTCI purchase decision. The individual faces the insurable LTC risk of a chronic illness. There are two types of care one can receive, family care or nursing home care. The type of care received depends on the availability, price, and preferences for each type of care; having long-term care insurance increases the availability and decreases the price of the insured type of LTC. Individuals buy LTCI when the benefits of the policy, in discounted utility terms, outweigh the reduction of income in the present, i.e. when lifetime expected utility is increased. The model was created to focus on the role of adult children as potential caregivers and on nursing homes as the only non-family option. A key conclusion was that the presence of adult children as potential caregivers reduces the value of LTCI when the potential purchaser prefers care from children. However, the individual’s purchase decision was not modeled in the context of a couple. Qualitative work suggests that couples consider LTC planning and purchases of LTCI together (Stum, 2008, Sperber et al., 2014), raising a number of conceptual and empirical difficulties.

Moving to a couple-level framework entails four key changes from the individual model: (1) the decision; (2) the price; (3) the availability of family care and (4) the utility function. We discuss each of these four unique components next.

#### Decision

Instead of just a binary insure/not insure decision, a couple has 4 outcomes to choose from: insure no one, insure the husband, insure the wife, or insure both members.

#### Price

Couple-level insurance products also change the pricing of insuring both members, as joint policies are typically cheaper than the sum of two policies, although they are also are overall less generous than two individual policies, as the members typically share the maximum benefit amount.

#### Family care availability

A couple-level model must account for the fact that family care could be provided by a spouse, not just an adult child. This intra-family source of self-insurance potentially lowers the overall financial risk of a disability for a couple compared to a single individual and decreases the demand for formal long-term care services. Costa-Font & Courbage (2015) refer to this as ‘family bailout.’ However, the ability to self-insure within a couple depends on each spouse’s health and their ability and willingness to provide care. Therefore, couples must predict not just the likelihood of individual disability, but the *joint* distribution of the disability and mortality trajectories of both members of the couple.

#### Utility function/bargaining power

Moving to a couple-level model means changing from a unitary to either a cooperative or non-cooperative model of household decision-making, which introduces the potential for intra-household dynamics to play a role in the LTCI purchase decision. If one person is the financial decision-maker, responsible for the insurance decision, they may not put the same amount of weight on the other person’s preferences or on the outcomes that may occur after the decisionmaker’s own death. This scenario could be due to non-cooperative elements of the model of marriage (Becker, 1973, Becker, 1981, Grossbard, 1984). Gender-based resource control, related to gender balance theory, has been demonstrated to influence a variety of consumption and investment decisions in several countries (Schmeer, 2005, Gummerson & Schneider, 2013, Opata et al., 2020, Quisumbing & Maluccio, 2003, Basu & Maitra, 2020, Booysen & Guvuriro, 2021, Menon et al., 2018).

### Model predictions

Brown & Finkelstein (2007a) highlight that moving to a couple-model could increase or decrease the couple’s willingness to pay for LTCI. For example, expected reliance on the spouse for LTC may decrease willingness to pay for LTCI, whereas desire to preserve assets for a surviving spouse may increase it. Thus, economic theory does not lead to predictions on the overall level of insurance within a couple compared to predictions from a unitary model, only that it might be different. Indeed, since the demand for formal LTC is a function of the *joint* distribution of the disability and mortality trajectories of both spouses, and of individual preferences which are largely unobserved, making a priori predictions of LTCI coverage is difficult.

Changes in the pricing regime over our study period, however, make some predictions possible. If couples have no individual- or couple-specific information, they should be more likely to purchase for the woman prior to April 2013, when unisex pricing was offered. Women have a much higher risk of using nursing home care and when LTCI prices were unisex, purchasing a LTCI policy for the woman was a better deal on average (Brown & Finkelstein, 2007a). After sex-specific pricing was introduced, this preference to purchase for women should decrease, and perhaps disappear if the underwriting correctly reflects the risk profile.

Second, we hypothesize that women’s bargaining power would be associated with higher LTCI coverage for women. Since a woman typically has a longer life expectancy than her husband, she may care more about preserving wealth over a longer time horizon or may value her LTC options after the man dies more than her spouse, increasing the probability of insuring herself, all else equal.

## Methods

### Data

We use data from twelve waves of the *Health and Retirement Study (HRS)* 1996–2018, a nationally representative bi-annual survey of middle-age and older adults in the U.S. The HRS is sponsored by the National Institute on Aging (grant number NIA U01AG009740) and is conducted by the University of Michigan. We use the RAND HRS Family and Longitudinal Files (RAND, 2022; RAND, 2023). Respondents were ages 51–61 when they entered the sample initially, or the spouse of the selected respondent, and are followed over time, thus they are old enough to have formed expectations and decisions about LTC planning and LTCI purchase. We merge the publicly available data with the Cross-Wave Geographic Information (State) file to match respondents to their state of residence, obtained through the MICDA secure computing environment (Health and Retirement Study, 2022).

### Sample

We use observations from married opposite-sex couples in which both members appear in the HRS data for at least two consecutive waves, and both answered the LTCI questions in these waves (See Appendix Table 4 for sample restrictions and size). We focus on the couple’s initial LTCI purchase decision (Konetzka & Luo, 2010) since it seems to be the decision that matters; only 2% of couples in our data eventually report buying a policy for the other spouse at a later date. We limit the sample to couples who both report not having private LTCI or government LTCI (through VA or Medicaid) in the first year of any two-year transition.

To assess eligibility for private LTCI, we apply typical underwriting standards, defining an individual as potentially eligible if they have fewer than two activities of daily living (ADL) limitations, no history of stroke or nursing home use by time *t*. In addition, the individual must be 78 or under at time *t*.^1^ We then examine the LTCI purchasing patterns of couples where at least one member is eligible and compare patterns to the subset of couples where both members are eligible.

Our main analyses require that both members of the couple are eligible to purchase a private LTCI policy. The potential purchaser sample comprises of 26,951 couple-wave observations, representing 7867 unique couples (Table 1). If one member of a couple dies or is no longer eligible to purchase LTCI, the couple leaves the sample. We drop respondents from VT and UT due to small sample sizes. For an examination of the regulatory change that allowed for sex-specific pricing rather than unisex pricing of private LTCI, we further drop respondents from CO and MT, since they had no change in the pricing regulation.

**Table 1:** LTCI Purchase Patterns for Married Individuals

Purchase Pattern	(1) At least One Member Eligible (%)	(2) Both Members Eligible (%)	(3) Both members eligible; woman decision maker (%)
N (couples)	8772	7867	646
Purchases in time t + 1			
No purchase	93.30	93.31	92.33
Purchase for man only	2.49	2.47	2.46
Purchase for woman only	2.61	2.52	3.72
Purchase for both	1.61	1.70	1.49
In Time t + 1 to t + 10 (Couple Observations)			
NEVER purchase between t + 1 and t + 10	67.57	67.61	59.60
Purchased for man only	9.95	9.98	10.37
Purchased for woman only	11.17	10.98	15.63
Purchased for both	11.31	11.43	14.40
Purchase Patterns under Unisex Pricing (1996–2012)			
N (couples)			
No purchase	93.21	93.20	92.63
Purchase for man only	2.47	2.48	2.37
Purchase for woman only	2.54	2.45	3.20
Purchase for both	1.78	1.87	1.81
Purchase Patterns under Sex-Specific Pricing (2013–2018)			
N (couples)			
No purchase	93.81	94.00	91.13
Purchase for man only	2.61	2.41	2.83
Purchase for woman only	3.00	2.95	5.85
Purchase for both	0.59	0.64	–

suppressed due to sample size

### Key measures

#### Dependent variable

We use the question from the HRS: “Not including government programs, do you now have any LTCI which specifically covers nursing home care for a year or more or any part of personal or medical care in your home?” To be coded as a purchaser, an individual responded “no” in time *t* and “yes” in *t* + *1*. We then assess the purchase status of their spouse. We code purchase status as being neither member of the couple bought insurance, the couple bought insurance for the woman, the couple bought insurance for the man, or the couple bought insurance for both members (bought for no one is the reference).

#### Key independent variable: Bargaining power

To measure the relative bargaining power of the female in the relationship, we use survey responses to the question “Who has the final say in decisions in your household? … What health insurance to buy?” The woman is coded as the financial decision-maker if she responded “I do always” or “I do mostly” in time t, regardless of what her spouse answers. We conduct a robustness analysis among couples in which both agree that the woman has decision-making power compared to couples who both agree the man has the final decision on health insurance decisions, dropping the cases where the man and woman disagree.

To illuminate whether the effects are related to decision-making power or simply financial awareness and information, we assess the robustness of our findings by using an alternative measure of the woman’s involvement in household finances. In the HRS, one person in a couple is designated as the financial respondent to the survey, and that person then answers the detailed financial questions. This should be the person who is most familiar with the household finances. As an alternative definition, we re-estimate the model but instead include an indicator for whether the female was designated as the financial respondent.

#### Control variables

To isolate the effect of female bargaining power, we include additional controls that have been shown to be important determinants of individual demand and supply for LTCI in the literature. Some of these are measured at the household level, while others are at the individual level and thus have values for both the male and the female.

#### Household financial considerations

Models include total household income, indicators for the quartiles of net worth (adjusted for inflation using the CPI, reported in constant 2018 dollars), and an indicator for home ownership. Further, we include an indicator for whether the couple has a will or a trust to indicate their financial planning horizon.

#### Household’s shared potential family care

To control for the potential sources of non-spousal family care to either member of the couple, we include a count of the number of children under 18 in the household and an indicator for any co-residential children ages 19+. The former could indicate additional household members in need of care, and the latter that an additional caregiver (or care recipient) is available. To control for experience with LTC (Coe et al., 2015), we include an indicator of whether either member of the couple had provided family care in the past. Beyond experience gained from providing family care in the past, care to a parent may reflect willingness to provide care to a spouse when needed, or knowledge of holes in LTC coverage under existing public insurance systems.

#### Household’s differential sources of family care

To control for the differential potential sources of family care between members of the couple, we include indicator variables for whether either member of the couple has step-children, as well as indicators for whether there are gender-concordant children (i.e., the man has sons; the woman has daughters) (Pezzin & Schone, 1999).

#### Demographics

Models include individual age, education, and race. Because of a high degree of correlation in these demographics within a couple, we have coded these as levels for the men in the sample, and as relative categories for the women. For example, the man’s education is defined as a categorical variable (Less than HS (ref), GED or HS degree, at least some college degree), and the woman’s education as an indicator for whether she has at least as much education as her husband. The man’s race (White (ref), African American, or other race) is in the model, while an indicator for the wife having a different race than the husband is included. The man’s age (continuous) and a variable capturing the difference in the ages between the man and woman is also included.

#### Health

We include individual self-reported fair or poor health, dichotomized from a 5-point Likert scale, and whether the individual currently smokes, separately for both the man and woman.

#### Risk aversion

There are several questions in the HRS used to capture an individual’s risk aversion. Because some measures show little variation in our sample (ie seatbelt wearing), or high degrees of missingness (gambles), here we use the sum of several health-related preventative services an individual could receive. For men, it is the sum of indicators for cholesterol tests, prostate exams, and flu shot receipt; for women it is the sum of indicators for pap smears, mammograms, cholesterol tests, and flu shot receipt.

### Models

#### LTCI purchase analysis

We estimate the following multinomial logit purchase model:

1 $${{LTCI}}_{t+1}={\beta }_{0}+{{\beta }_{1}{C}_{t}+\beta }_{2}{X}_{t}+{{\beta }_{3}R}_{t}+{{s}_{t}{+\gamma }_{t}+\varepsilon }_{c,t}$$

We define LTCI to be the four categories describing the couple’s purchase behavior in t + 1: for neither (ref); for man only; for woman only; and for both. We focus on ${\beta }_{1}$, which measures how LTCI purchase decisions vary with bargaining power (C_t_), The other demand-side factors (X_t_) included are as described in the measures section above. We also include controls for supply-side factors. ${R}_{t}$ is a binary indicator that is coded as 1 for 2013–2018 and 0 for 1996 to 2012, indicating the change in the sex-specific pricing regime. State (${s}_{t}$) fixed-effects account for non-time-varying differences across states to help adjust for state-level insurance regulatory regimes. Year (γ_t_) fixed effects control for common time trends. We use a multinomial logit to estimate Eq. 1; standard errors are clustered at the couple level. We report marginal effects calculated as the derivative of the response with respect to the variable of interest averaged across the sample, or the average marginal effect (AME).

Beyond controlling for the change to sex-specific pricing for private LTCI introduced during the study period in the model, we also examine the combination of bargaining power of the woman and this policy change (${R}_{t}$). We re-estimate a model very similar to Eq. 1 but that includes an interaction term between whether the woman has the final say in what health insurance to buy and a time indicator of the pricing policy change ${R}_{t}$.

We rerun the main model (Eq. (1)) exploring whether the woman being the financial respondent matters. Being the financial respondent likely indicates that the woman has detailed knowledge about the couple’s finances (and potentially more than the man), but being a financial respondent may not reflect whether she has the power to decide which health insurance plan to purchase.

Finally, given that one of the goals of this study is to understand purchasing for just one couple member, and which member the couple insures, we perform a conditional analysis, estimating the main model (Eq. (1)) on the sub-sample of couples insuring only one member. In this examination, the outcome measure (LTCI_t+1_) becomes a binary indicator for purchasing for the woman and not the man (and the reverse), and we estimate the model with a logit. We report the average marginal effect.

#### Sensitivity analysis

The data provides individual reports of who is the decision maker for health insurance. In sensitivity analysis, we exclude couples in which the man disagrees with the woman about who is the decision maker for health insurance.

## Results

### Descriptive results

#### LTCI purchase patterns

While in any given year most couples do not buy LTCI (93.3%), almost one-third of couples ever purchase LTCI while in the panel among those in which at least one member meets our definition of LTCI purchase eligibility in time t (Table 1; Column 1). The most common purchase pattern was for one member of a couple exclusively, at 21.1% combined, while only 11% bought for both members of the couple. This pattern is not explained by differential spousal eligibility because this purchase pattern persists in the subsample where both members of the couple meet our LTCI purchase eligibility criteria in time t (Table 1; Column 2). However, differences begin to emerge when examining the couples where women have bargaining power (Table 1; Column 3). Overall, more couples buy insurance (40% vs. 33%) when the woman has bargaining power, and these additional purchases are more concentrated in buying for the woman only (16% vs. 11%) or for both members of the couple (14% vs. 11%).

Next, we examine purchasing patterns under unisex pricing vs. sex-specific pricing regimes. As expected, we find that among couples who are both eligible (column 2), more women are insured in the earlier time period (2.45 + 1.87 vs. 2.95 + 0.64), with the major difference coming from the number of couples who are both insured. Among couples in which both are eligible and women have more bargaining power, we find that, counter to our hypothesis, more insurance is purchased in the later period with higher prices for women (8.9% vs. 7.4%). This increase is occurring among couples deciding to insure only one member of the couple, but the increase is in both coverage of men and coverage of women.

#### Sample characteristics by purchase status

There are several apparent differences by LTCI purchase status between couples who purchased for one member or neither and couples who purchased for both (Table 2). Consistent with previous findings on individual purchase (Brown & Finkelstein, 2007b, Courtemanche & He, 2009, Konetzka, 2014), couples who purchased for both members are considerably wealthier and have higher home ownership rates compared to couples who buy for only one member or never buy. They are also more likely to have a will or trust, to be better educated and less likely to smoke. Education differentials appear to be correlated with purchase outcomes; couples in which the woman is at least as educated as the man are more likely to purchase for both.

**Table 2:** Summary statistics measured at the wave prior to LTCI purchase decision among couples who are both LTCI eligible, by purchase decision and bargaining power of the woman

	Total Sample				Woman is the Decision Maker			
	Purchased for neither	Purchased for man only	Purchase for woman only	Purchased for both	Purchased for both	Purchased for man only	Purchase for woman only	Purchased for both
*Couple-dynamics*								
Woman has the final say of what health insurance to buy	0.173	0.169	0.278	0.180	1	1	1	1
Woman is the financial respondent	0.368	0.344	0.413	0.395	0.562	0.588	0.575	0.567
*Financial Considerations*								
Total household income (in 2018 dollars/thousands)	116.3	125.1	138.1	160.0	109.7	127.6	134.2	167.6
Net worth (1st quartile)	0.0976	0.103	0.0972	–	0.115	0.118	0.113	–
Net worth (2nd quartile)	0.219	0.258	0.257	–	0.265	0.176	0.300	–
Net worth (3rd quartile)	0.321	0.318	0.323	0.293	0.335	0.431	0.250	0.400
Net worth (4th quartile)	0.363	0.321	0.323	0.551	0.284	0.275	0.338	0.533
Owns a home	0.924	0.930	0.927	0.958	0.914	0.980	0.925	1
Has a will/trust	0.573	0.570	0.556	0.802	0.471	0.608	0.588	0.867
*Shared Family Care Measures*								
Number of children (<18) in household	0.134	0.142	0.177	0.0778	0.183	0.0784	0.237	0.0667
Couple has coresidential children 19 +	0.244	0.315	0.257	0.120	0.276	0.353	0.250	0.0667
Couple has experience providing LTC	0.512	0.517	0.556	0.437	0.527	0.569	0.525	0.633
*Demographics*								
Man is White	0.844	0.808	0.736	0.904	0.769	0.745	0.688	0.900
Man is African American	0.0910	0.129	0.181	0.0659	0.123	0.118	0.250	0.100
Man is other race	0.0650	0.0629	0.0833	0.0299	0.108	0.137	0.0625	0
Man has at least some college degree	0.535	0.586	0.535	0.635	0.433	0.608	0.450	0.567
Man has GED or high school degree	0.333	0.315	0.323	0.293	0.387	0.235	0.325	0.300
Man has less than HS degree	0.132	0.0993	0.142	0.0719	0.180	0.157	0.225	0.133
Man’s age	62.89	61.16	62.02	61.89	62.16	61.98	62.44	61.53
Woman is not the same race	–	–	–	–	–	–	–	–
Woman is at least as educated as man	0.812	0.838	0.809	0.850	0.858	0.863	0.912	0.933
Relative age (Man’s age-woman’s age)	3.017	3.487	3.451	2.617	3.519	4.176	5.150	3.333
*Health*								
Man currently smokes	0.113	0.159	0.0938	0.0539	0.163	0.157	0.100	0
Man reported fair/poor health	0.133	0.129	0.139	0.144	0.180	0.137	0.163	0.167
Woman currently smokes	0.0872	0.116	0.108	0.0778	0.0992	0.0980	0.0875	0.0667
Woman reported fair/poor health	–	–	–	–	–	–	–	–
*Risk Aversion*								
Man’s Risk Aversion Score	2.089	2.050	2.052	2.287	1.983	1.902	2.050	2.033
Woman’s Risk Aversion Score	2.894	2.838	2.913	3.096	2.890	2.902	2.900	3.300
*Differential Family care sources*								
Man has stepchildren	0.200	0.262	0.247	0.120	0.218	0.196	0.300	0.200
Woman has stepchildren	0.270	0.371	0.372	0.246	0.296	0.412	0.563	0.267
Man has sons	0.853	0.864	0.868	0.844	0.861	0.922	0.887	0.967
Woman has daughters	0.827	0.844	0.816	0.790	0.834	0.843	0.838	0.667
Observations (couple-waves)	11009	302	288	167	1906	51	80	30

All variables are at the household level and unweighted. Income is reported in 2018 dollars, adjusted for inflation using the Consumer Price Index (CPI). All individual-level variables have been recoded to represent whether either partner has the characteristic (e.g., whether either partner has a will/trust, whether either partner’s mother is still living, etc.)

suppressed due to sample size

When it comes to the bargaining power within a couple, couples in which the woman is the final decision-maker of what health insurance to buy are more likely to insure the woman only. Family LTC care provision is highly correlated with buying for both members of the family, but only for those couples with a female decision-maker.

Our measures of differential sources of family care are largely not strongly correlated with LTCI purchase decisions in the overall sample. However, among those with a female decision-maker, the woman having step-children is correlated with purchase, especially purchasing only for the woman.

Availability of care/care responsibilities are highly correlated with purchase decisions. Very few couples with young children in the household insure both members of the couple; for families with female decision-makers, they are also much less likely to insure the man only. The presence of older coresidential children is correlated with lower likelihood of buying for both members, especially for families with female decision-makers.

Health measures seem to be less strongly correlated with LTCI purchase decisions except for male smoking behavior. Couples in which the men currently smoke are more likely to purchase LTCI for the man only, and less likely to purchase for both members of the couple. This is consistent with the woman as the decision-maker, although then they are more likely to forego LTCI altogether. Risk aversion is correlated with higher prevalence of LTCI purchase in general.

### Main results

Table 3 presents our main results of interest. Each panel represents a different regression model; full results are presented in Appendix Tables 5–8; here we present the marginal effects of interest measuring women’s bargaining power. In a multivariable regression model, we find that a woman having bargaining power is correlated with a higher likelihood of purchasing LTCI for the woman (Panel A). This is especially pronounced once sex-specific pricing is introduced (Panel B), when the price for insuring women presumably increased. Specifically, compared to the unisex pricing period, couples are significantly less likely to insure both in the sex-specific pricing period, but the effect is slightly larger among couples in which the woman decides what health insurance to buy. In Panel C, we experiment to see if there are different relationships between being the financial respondent, thus presumably having more knowledge about the couple finances, and being a decision-maker. Indeed, we find that being a financial respondent is not correlated with LTCI decisions.

**Table 3:** Results from Multinomial Logit Model of LTCI Purchase – Both Members of Couple ‘Eligible’ By Our Criteria (reference is not purchased for either)

	Purchased for man only	Purchased for woman only	Purchased for both
*Panel A: Full Sample*			
Woman has the final say about the health insurance to buy	−0.00319	0.0135^***^	0.00316
	(0.00410)	(0.00368)	(0.00300)
*N*	11,766	11,766	11,766
*Panel B: Woman has the final say about the health insurance to buy interacts with the pre and post 2013 indicator indicating sex-specific pricing began*			
Woman has the final say: sex-specific pricing vs. unisex pricing	0.0210	0.0268	−0.211^***^
	(0.0249)	(0.0312)	(0.00335)
Woman doesn’t have the final say: Sex-specific pricing vs. unisex pricing	0.00423	0.00301	−0.206^***^
	(0.0159)	(0.0120)	(0.00117)
*N*	11,357	11,357	11,357
*Panel C: Robustness check: Definition*			
Woman is the financial respondent	−0.00339	0.00360	0.00476
	(0.00357)	(0.00320)	(0.00246)
*N*	11,758	11,758	11,758
*Panel D: Conditional Analysis: Couple purchased private LTCI but only for the woman*			
Woman has the final say about the health insurance to buy		0.141^**^	
		(0.0492)	
*N*		579	

We present marginal effects from multinomial logistic regressions; standard errors are reported in parentheses. Models are unweighted. All models excluded state UT and VT. Panel A, B, D, E controlled for state fixed effects, wave fixed effects and an indicator for year > =2013. Panel C controlled for wave fixed effects and an indicator for year > =2013. Panel E was run on the Panel A’s sample and excluded ME

Panel B also dropped CO and MT

Significance denoted as follows: *p < 0.05, **p < 0.01, ***p < 0.001

In Panel D, we take a deeper dive to understand the factors that are correlated with buying for only one member of the couple. Among the sample where the final decision was to only insure one member of the couple, intra-couple bargaining remains important. If the woman is a decision-maker, there is 1.4 percentage point increase in the likelihood of the woman being the insured member of the couple; indeed, it is the second largest positive predictor of the woman having LTCI in this sample.

#### Sensitivity analysis

Results are robust to excluding couples in which the man disagrees with the woman that she has the final say about which health insurance to purchase (Appendix Table 9).

## Discussion

We contribute to the literature on LTCI decisions among older adults by describing purchase patterns of LTCI for couples and examining how female bargaining power is associated with the observed purchase patterns. We find that couples who purchased LTCI were equally likely to insure the man exclusively, the woman exclusively, or the couple. The observed pattern of relatively equal likelihood of purchase for the man, for the women, and for both members of the couple, is not explained by spouse ineligibility. While individual-level factors previously explored, such as financial factors, alternative sources of family care, and future expected need were associated with a higher likelihood of purchase, they are correlated with both members of the couple getting insurance and do not help explain differential insurance coverage within the couple. Even the advent of sex-specific pricing did not sway patterns substantially, a time after which purchasing for the woman would presumably become more expensive relative to the man. We find that for the 16% of eligible couples who bought a LTCI policy prior to April 2013, purchase patterns did not meet a priori expectations that married women would be more likely to be covered than married men based on the relative value of the insurance (Brown & Finkelstein, 2007b).

The purchasing story is much more nuanced within couples and includes who has decision-making power over these types of insurance decisions within the couple. A woman having decision-making power about insurance decisions increases the likelihood of purchasing for the woman exclusively and for the women as a part of joint purchase. These findings are consistent with the literature that has repeatedly found that there are gendered spending patterns depending on who has control over the funds within the household, and that this gendered spending increases spending on women and children (Schmeer, 2005, Gummerson & Schneider, 2013, Opata et al., 2020, Quisumbing & Maluccio, 2003, Basu & Maitra, 2020, Booysen & Guvuriro, 2021). The desire to insure the female member of the household could signal a stronger desire to maintain access to a wide variety of LTC options for one’s own LTC needs later in life after the husband dies, a desire to decrease family caregiving burdens on adult children, or a stronger bequest motive to children among women compared to men.

This paper has several limitations. Survey responses about LTCI purchase may be subject to measurement error. Consistent with our research questions, our analysis is descriptive in nature and does not have causal interpretations, although we controlled for a rich set of variables available in HRS. We cannot observe how an individual was offered LTCI, i.e., through an employer, which could influence who buys the policy. Our measure of eligibility for LTCI is likely imperfect compared to the health assessment done during the underwriting process prior to purchase, thus could involve some misclassification. Finally, as in much survey-based research, most of our measures of interest from the HRS are proxies for the concept we want to capture.

This paper offers a descriptive view of factors that are associated with the observed LTCI purchase patterns of married couples, which had not previously been described using nationally representative data of middle-aged to older adults. Efforts to expand risk coverage among older Americans should consider that couples are more likely to insure only one member of the household than they are to insure both members, even when both are eligible. Additionally, lack of financial decision-making power is rarely considered as a barrier to LTCI for older women. Our results suggest that women desire more LTCI than they can get in the private market when they lack of bargaining power within their household. Even when there was unisex pricing, essentially giving a discount to the cost of insuring women (Brown & Finkelstein, 2007b), there remained different purchasing patterns within households based on the sex of the decision-maker. This work provides yet another reason for low demand for LTCI, but not one easily remedied by the market.

## Data Availability

The Health and Retirement Study is available for download https://hrsdata.isr.umich.edu/.

## Appendix

Tables 4–9

**Table 4:** Sample Restrictions

Individual-Level Restrictions	Individual-wave
Married individuals	138,919
Removing individuals with no spouse present	133,440
Removing same-sex couples	133,230
Couple-Level Restrictions	Couple-wave
Removing waves with missing LTCI information for either partner in a couple	64,127
Responses from 2+ consecutive waves	49,857
Neither has LTCI in the base wave (time t)	41,566
No government insurance in the base wave (time t)	35,938
Both are under 79 in the base wave (time t)	31,773
Both eligible for LTCI in the base wave (time t)	26,953
Removing if only one observation in a given state	26,951

Eligibility is defined as zero or one ADL limitation, and no history of nursing home stay and no history of stroke

**Table 5:** Full Results from Primary Model: Multinomial Logit Model of LTCI Purchase – Both Members of Couple ‘Eligible’ (reference is not purchased for either)

	Purchased for man only	Purchased for woman only	Purchased for both
*Couple-dynamics*			
Woman has the final say about the health insurance to buy	−0.00319	0.0135^***^	0.00316
	(0.00410)	(0.00368)	(0.00300)
*Financial Considerations*			
Total household income (in 2018 dollars/thousands)	0.00000380	0.0000231^**^	0.00000318
	(0.0000104)	(0.00000782)	(0.00000385)
Net worth (1^st^ quartile)	0.00667	0.0000799	−0.0107
	(0.00716)	(0.00784)	(0.00660)
Net worth (2^nd^ quartile)	0.00637	0.00389	−0.00824^*^
	(0.00458)	(0.00469)	(0.00395)
Net worth (3^rd^ quartile)	0.00216	0.00310	−0.00603^*^
	(0.00391)	(0.00387)	(0.00279)
Owns a home	0.00867	0.00257	−0.00562
	(0.00777)	(0.00740)	(0.00614)
Has a will/trust	0.00448	0.00351	0.00888^**^
	(0.00367)	(0.00383)	(0.00323)
*Shared Family Care Measures*			
Number of children (<18) in household	−0.00628	0.000818	−0.00434
	(0.00341)	(0.00336)	(0.00386)
Couple has coresidential children 19 +	0.00822^*^	0.000517	−0.00903^*^
	(0.00348)	(0.00363)	(0.00358)
Couple has experience providing LTC	0.00121	0.00424	−0.00154
	(0.00320)	(0.00328)	(0.00227)
*Demographics*			
Man is African American	0.00793	0.0184^***^	0.00133
	(0.00478)	(0.00507)	(0.00502)
Man is Other race	−0.0106	0.0159^*^	−0.00901
	(0.00753)	(0.00667)	(0.00685)
Man has at least some college degree	0.0136^*^	−0.00466	0.00730
	(0.00600)	(0.00549)	(0.00465)
Man has GED or high school degree	0.00822	−0.00233	0.00495
	(0.00585)	(0.00545)	(0.00466)
Man’s age	−0.000730^*^	−0.000417	−0.000467^*^
	(0.000284)	(0.000262)	(0.000202)
Woman is not the same race	0.0234^***^	−0.00750	0.00993
	(0.00579)	(0.00728)	(0.00521)
Woman is at least as educated as man	0.00648	−0.00331	0.00288
	(0.00439)	(0.00435)	(0.00330)
Relative age (Man’s age-Woman’s age)	0.000512	0.000218	−0.000242
	(0.000391)	(0.000341)	(0.000240)
*Health*			
Man currently smokes	0.00628	−0.00982	−0.00715
	(0.00484)	(0.00546)	(0.00483)
Man reported fair/poor health	−0.000942	−0.000194	0.00873^**^
	(0.00478)	(0.00443)	(0.00330)
Woman currently smokes	0.00386	0.00792	0.00242
	(0.00505)	(0.00585)	(0.00426)
Woman reported fair/poor health	0.00165	−0.000629	−0.00472
	(0.00453)	(0.00460)	(0.00429)
*Risk Aversion*			
Man’s Risk Aversion Score	0.00158	−0.0000849	0.00210
	(0.00175)	(0.00169)	(0.00150)
Woman’s Risk Aversion Score	−0.000765	0.000728	0.000784
	(0.00143)	(0.00159)	(0.00107)
*Differential Family care sources*			
Man has stepchildren	0.00211	0.000439	−0.00881^*^
	(0.00443)	(0.00430)	(0.00396)
Woman has stepchildren	0.00834	0.00760	0.00230
	(0.00436)	(0.00417)	(0.00324)
Man has sons	0.00162	−0.000116	0.000506
	(0.00498)	(0.00518)	(0.00320)
Woman has daughters	0.00251	−0.00411	−0.00197
	(0.00441)	(0.00451)	(0.00285)
*N*	11766	11766	11766

We present marginal effects from multinomial logistic regressions; standard errors are reported in parentheses. Models are unweighted. All models excluded state UT and VT, and controlled for state fixed effects, wave fixed effects and an indicator for year > =2013

Significance denoted as follows: **p* < 0.05, ***p* < 0.01, ****p* < 0.001

**Table 6:** Full Results from Multinomial Logit Model of LTCI Purchase – Both Members of Couple ‘Eligible’ (reference is not purchased for either): Unisex-Pricing (1996–2012) vs. Gender-Specific Pricing (2013–2018)

	Purchased for man only	Purchased for woman only	Purchased for both
*Couple-dynamics*			
Woman has the final say about the health insurance to buy	−0.00150	0.0157^**^	0.00406
	(0.00390)	(0.00493)	(0.00361)
*Financial Considerations*			
Total household income (in 2018 dollars/thousands)	0.00000436	0.0000234^**^	0.00000346
	(0.0000104)	(0.00000772)	(0.00000391)
Net worth (1^st^ quartile)	0.00615	−0.00113	−0.0107
	(0.00718)	(0.00799)	(0.00667)
Net worth (2^nd^ quartile)	0.00442	0.00499	−0.00828^*^
	(0.00453)	(0.00468)	(0.00401)
Net worth (3^rd^ quartile)	0.00159	0.00316	−0.00625^*^
	(0.00395)	(0.00388)	(0.00284)
Owns a home	0.00987	0.000661	−0.00581
	(0.00775)	(0.00729)	(0.00623)
Has a will/trust	0.00382	0.00392	0.00855^**^
	(0.00369)	(0.00388)	(0.00324)
*Shared Family Care Measures*			
Number of children (<18) in household	−0.00613	0.000441	−0.00441
	(0.00343)	(0.00350)	(0.00392)
Couple has coresidential children 19 +	0.00673	0.000548	−0.00893^*^
	(0.00351)	(0.00365)	(0.00359)
Couple has experience providing LTC	0.00238	0.00422	−0.00169
	(0.00324)	(0.00329)	(0.00233)
*Demographics*			
Man is African American	0.00799	0.0181^***^	0.00123
	(0.00477)	(0.00502)	(0.00513)
Man is Other race	−0.0101	0.0158^*^	−0.00802
	(0.00776)	(0.00664)	(0.00687)
Man has at least some college degree	0.0119^*^	−0.00448	0.00755
	(0.00594)	(0.00548)	(0.00472)
Man has GED or high school degree	0.00734	−0.00353	0.00496
	(0.00579)	(0.00547)	(0.00474)
Man’s age	−0.000729^*^	−0.000469	−0.000447^*^
	(0.000290)	(0.000262)	(0.000203)
Woman is not the same race	0.0231^***^	−0.0112	0.00823
	(0.00592)	(0.00757)	(0.00530)
Woman is at least as educated as man	0.00520	−0.00390	0.00259
	(0.00440)	(0.00440)	(0.00337)
Relative age (Man’s age-Woman’age)	0.000456	0.000226	−0.000241
	(0.000401)	(0.000349)	(0.000248)
*Health*			
Man currently smokes	0.00601	−0.00871	−0.00848
	(0.00488)	(0.00551)	(0.00519)
Man reported fair/poor health	−0.00404	0.000498	0.00832^*^
	(0.00495)	(0.00451)	(0.00332)
Woman currently smokes	0.00338	0.00780	0.00165
	(0.00515)	(0.00590)	(0.00451)
Woman reported fair/poor health	0.00290	−0.000637	−0.00455
	(0.00447)	(0.00467)	(0.00435)
*Risk Aversion*			
Man’s Risk Aversion Score	0.00135	0.000457	0.00209
	(0.00178)	(0.00171)	(0.00155)
Woman’s Risk Aversion Score	−0.00128	0.000596	0.000729
	(0.00144)	(0.00161)	(0.00110)
*Differential Family care sources*			
Man has stepchildren	0.00103	−0.00105	−0.00884^*^
	(0.00455)	(0.00430)	(0.00399)
Woman has stepchildren	0.00833	0.00785	0.00272
	(0.00449)	(0.00425)	(0.00334)
Man has sons	0.00137	0.00155	0.000396
	(0.00498)	(0.00532)	(0.00327)
Woman has daughters	0.00338	−0.00416	−0.00137
	(0.00452)	(0.00458)	(0.00296)
*Marginal effects for the interaction term between female has the final say and the 2013 indicator*			
Woman has the final say post 2013 compared with pre 2013 period	0.0210	0.0268	−0.211^***^
	(0.0249)	(0.0312)	(0.00335)
Woman doesn’t have the final say post 2013 compared with pre 2013 period	0.00423	0.00301	−0.206^***^
	(0.0159)	(0.0120)	(0.00117)
*N*	11357	11357	11357

We present marginal effects from multinomial logistic regressions; standard errors are reported in parentheses. Models are unweighted. All models excluded state UT and VT, CO and MT and controlled for wave fixed effects and an indicator for year > =2013

Significance denoted as follows: **p* < 0.05, ***p* < 0.01, ****p* < 0.001

**Table 7:** Full Results from Multinomial Logit Model of LTCI Purchase – Both Members of Couple ‘Eligible’ (reference is not purchased for either)

	Purchased for man only	Purchased for woman only	Purchased for both
*Couple-dynamics*			
**Woman is the financial respondent**	−0.00339	0.00360	0.00476
	(0.00357)	(0.00320)	(0.00246)
*Financial Considerations*			
Total household income (in 2018 dollars/thousands)	0.00000355	0.0000236^**^	0.00000387
	(0.0000105)	(0.00000774)	(0.00000384)
Net worth (1^st^ quartile)	0.00716	0.000478	−0.0110
	(0.00724)	(0.00780)	(0.00655)
Net worth (2^nd^ quartile)	0.00663	0.00410	−0.00844^*^
	(0.00463)	(0.00474)	(0.00392)
Net worth (3^rd^ quartile)	0.00231	0.00353	−0.00616^*^
	(0.00396)	(0.00385)	(0.00276)
Owns a home	0.00881	0.00477	−0.00588
	(0.00778)	(0.00751)	(0.00613)
Has a will/trust	0.00455	0.00308	0.00871^**^
	(0.00366)	(0.00383)	(0.00317)
*Shared Family Care Measures*			
Number of children (<18) in household	−0.00645	0.000991	−0.00417
	(0.00340)	(0.00339)	(0.00379)
Couple has coresidential children 19 +	0.00816^*^	0.000460	−0.00878^*^
	(0.00348)	(0.00362)	(0.00352)
Couple has experience providing LTC	0.00117	0.00421	−0.00149
	(0.00320)	(0.00328)	(0.00227)
*Demographics*			
Man is African American	0.00801	0.0187^***^	0.000948
	(0.00479)	(0.00513)	(0.00497)
Man is Other race	−0.0109	0.0169^*^	−0.00880
	(0.00749)	(0.00670)	(0.00678)
Man has at least some college degree	0.0132^*^	−0.00520	0.00846
	(0.00602)	(0.00557)	(0.00473)
Man has GED or high school degree	0.00794	−0.00236	0.00550
	(0.00586)	(0.00548)	(0.00467)
Man’s age	−0.000728^*^	−0.000446	−0.000471^*^
	(0.000285)	(0.000260)	(0.000198)
Woman is not the same race	0.0235^***^	−0.00666	0.00980
	(0.00577)	(0.00715)	(0.00519)
Woman is at least as educated as man	0.00654	−0.00296	0.00288
	(0.00439)	(0.00436)	(0.00327)
Relative age (Man’s age-Woman’s age)	0.000460	0.000359	−0.000185
	(0.000394)	(0.000349)	(0.000244)
*Health*			
Man currently smokes	0.00642	−0.0104	−0.00752
	(0.00483)	(0.00558)	(0.00480)
Man reported fair/poor health	−0.000862	0.000410	0.00882^**^
	(0.00479)	(0.00446)	(0.00324)
Woman currently smokes	0.00390	0.00693	0.00252
	(0.00504)	(0.00597)	(0.00428)
Woman reported fair/poor health	0.00159	−0.000365	−0.00462
	(0.00454)	(0.00460)	(0.00427)
*Risk Aversion*			
Man’s Risk Aversion Score	0.00156	−0.000589	0.00219
	(0.00175)	(0.00169)	(0.00149)
Woman’s Risk Aversion Score	−0.000784	0.000831	0.000772
	(0.00143)	(0.00158)	(0.00106)
*Differential Family Care Sources*			
Man has stepchildren	0.00227	0.000152	−0.00917^*^
	(0.00443)	(0.00432)	(0.00396)
Woman has stepchildren	0.00847	0.00796	0.00213
	(0.00440)	(0.00416)	(0.00326)
Man has sons	0.00156	−0.000779	0.000619
	(0.00499)	(0.00508)	(0.00321)
Woman has daughters	0.00240	−0.00427	−0.00178
	(0.00442)	(0.00457)	(0.00286)
*N*	11758	11758	11758

We present marginal effects from multinomial logistic regressions; standard errors are reported in parentheses. Models are unweighted. All models excluded state UT, VT, and ME. Models controlled for state fixed effects, wave fixed effects and an indicator for year > =2013. Models were run on Panel A’s sample (woman has the final say of what insurance to buy)

Significance denoted as follows: **p* < 0.05, ***p* < 0.01, ****p* < 0.001

**Table 8:** Full Results from the Conditional Analysis

	Purchased for man only	Purchased for woman only
*Couple-dynamics*		
Woman has the final say about the health insurance to buy	−0.148^**^	0.141^**^
	(0.0491)	(0.0492)
*Financial Considerations*		
Total household income (in 2018 dollars/thousands)	−0.000194	0.000276
	(0.000144)	(0.000147)
Net worth (1^st^ quartile)	−0.00100	−0.0319
	(0.0904)	(0.0912)
Net worth (2^nd^ quartile)	0.0335	−0.0632
	(0.0649)	(0.0659)
Net worth (3^rd^ quartile)	−0.0115	−0.00398
	(0.0533)	(0.0542)
Owns a home	0.0167	−0.00954
	(0.0939)	(0.0970)
Has a will/trust	0.0246	−0.00163
	(0.0489)	(0.0491)
*Shared Family Care Measures*		
Number of children (<18) in household	−0.0645	0.124
	(0.0516)	(0.0723)
Couple has coresidential children 19 +	0.0424	0.292^**^
	(0.0482)	(0.0965)
Couple has experience providing LTC	−0.0393	−0.185^*^
	(0.0452)	(0.0735)
*Demographics*		
Spouse is African American	−0.146^*^	−0.0662
	(0.0721)	(0.0735)
Spouse is Other race	−0.126	0.00246
	(0.0860)	(0.00391)
Spouse has at least some college degree	0.177	−0.264^**^
	(0.0968)	(0.0883)
Spouse has GED or high school degree	0.224^*^	−0.0933
	(0.0881)	(0.0597)
Spouse’s age	−0.00454	0.000873
	(0.00399)	(0.00404)
Woman is not the same race	0.228^**^	0.124
	(0.0862)	(0.0723)
Woman is at least as educated as man	0.0250	0.292^**^
	(0.0693)	(0.0965)
Relative age (Man’s age-Woman’s age)	−0.00511	−0.185^*^
	(0.00432)	(0.0735)
*Health*		
Spouse currently smokes	−0.0150	−0.155^*^
	(0.0784)	(0.0650)
Spouse reported fair/poor health	−0.0448	−0.0237
	(0.0638)	(0.0629)
Currently smokes	0.138^*^	0.0260
	(0.0649)	(0.0757)
Reported fair/poor health	0.00120	0.0155
	(0.0634)	(0.0641)
*Risk Aversion*		
Spouse’s Risk Aversion Score	−0.0158	−0.0214
	(0.0200)	(0.0226)
Risk Aversion Score	0.0228	0.0134
	(0.0231)	(0.0194)
*Differential Family care sources*		
Spouse has stepchildren	0.0136	−0.00659
	(0.0544)	(0.0582)
Has stepchildren	−0.000568	−0.00700
	(0.0601)	(0.0539)
Man has sons	0.000388	−0.00765
	(0.0613)	(0.0625)
Woman has daughters	0.0638	−0.0662
	(0.0609)	(0.0600)
*N*	578	579

We present marginal effects from multinomial logistic regressions; standard errors are reported in parentheses. Models are unweighted. All models excluded state UT and VT, and controlled for state fixed effects, wave fixed effects and an indicator for year > =2013

Significance denoted as follows: **p* < 0.05, ***p* < 0.01, ****p* < 0.001

**Table 9:** Results on Multinomial Logit Model of LTCI Purchase – Both Members of Couple ‘Eligible’ By Our Criteria, Allowing Subsequent LTCI Purchase (Drop couples with contradictions regarding who is the decision maker)

	Purchased for man only	Purchased for woman only	Purchased for both
*Couple-dynamics*			
Woman has the final say about the health insurance to buy	−0.00212	0.0106^**^	0.00321
	(0.00415)	(0.00357)	(0.00304)
*Financial Considerations*			
Total household income (in 2018 dollars/thousands)	0.00000360	0.0000203^*^	0.00000310
	(0.0000105)	(0.00000803)	(0.00000390)
Net worth (1^st^ quartile)	0.00679	−0.00312	−0.0107
	(0.00719)	(0.00802)	(0.00658)
Net worth (2^nd^ quartile)	0.00631	0.00249	−0.00816^*^
	(0.00462)	(0.00461)	(0.00391)
Net worth (3^rd^ quartile)	0.00206	0.00292	−0.00591^*^
	(0.00394)	(0.00379)	(0.00275)
Owns a home	0.00884	0.00148	−0.00572
	(0.00782)	(0.00757)	(0.00616)
Has a will/trust	0.00459	0.00268	0.00886^**^
	(0.00370)	(0.00376)	(0.00317)
*Shared Family Care Measures*			
Number of children (<18) in household	−0.00632	−0.000573	−0.00409
	(0.00342)	(0.00305)	(0.00381)
Couple has coresidential children 19 +	0.00844^*^	0.000199	−0.00885^*^
	(0.00350)	(0.00359)	(0.00351)
Couple has experience providing LTC	0.00116	0.00403	−0.00166
	(0.00323)	(0.00316)	(0.00228)
*Demographics*			
Man is African American	0.00808	0.0192^***^	0.00143
	(0.00482)	(0.00496)	(0.00508)
Man is Other race	−0.0108	0.0162^*^	−0.00891
	(0.00762)	(0.00656)	(0.00684)
Man has at least some college degree	0.0137^*^	−0.00574	0.00735
	(0.00604)	(0.00543)	(0.00462)
Man has GED or high school degree	0.00829	−0.00236	0.00505
	(0.00589)	(0.00538)	(0.00466)
Man’s age	−0.000746^**^	−0.000530^*^	−0.000443^*^
	(0.000286)	(0.000253)	(0.000197)
Woman is not the same race	0.0234^***^	−0.00667	0.00995
	(0.00585)	(0.00712)	(0.00517)
Woman is at least as educated as man	0.00642	−0.00391	0.00271
	(0.00442)	(0.00429)	(0.00329)
Relative age (Man’s age-Woman’s age)	0.000505	0.000352	−0.000241
	(0.000394)	(0.000335)	(0.000239)
*Health*			
Man currently smokes	0.00628	−0.00920	−0.00715
	(0.00487)	(0.00542)	(0.00481)
Man reported fair/poor health	−0.000954	−0.000223	0.00887^**^
	(0.00481)	(0.00442)	(0.00323)
Woman currently smokes	0.00376	0.00696	0.00231
	(0.00509)	(0.00576)	(0.00428)
Woman reported fair/poor health	0.00173	−0.000933	−0.00475
	(0.00456)	(0.00462)	(0.00428)
*Risk Aversion*			
Man’s Risk Aversion Score	0.00158	0.000704	0.00203
	(0.00177)	(0.00165)	(0.00149)
Woman’s Risk Aversion Score	−0.000790	0.000454	0.000697
	(0.00143)	(0.00157)	(0.00106)
*Differential Family care sources*			
Man has stepchildren	0.00241	−0.00139	−0.00834^*^
	(0.00445)	(0.00433)	(0.00389)
Woman has stepchildren	0.00848	0.00685	0.00239
	(0.00437)	(0.00417)	(0.00324)
Man has sons	0.00171	−0.00000787	0.000303
	(0.00502)	(0.00503)	(0.00321)
Woman has daughters	0.00230	−0.00277	−0.00219
	(0.00445)	(0.00439)	(0.00285)
*N*	11673	11673	11673

We present marginal effects from multinomial logistic regressions; standard errors are reported in parentheses. Models are unweighted. All models excluded state UT and VT, and controlled for state fixed effects, wave fixed effects and an indicator for year > =2013

Significance denoted as follows: **p* < 0.05, ***p* < 0.01, ****p* < 0.001

## Footnotes

1 While some studies of LTCI purchase have an upper age limit of 70, we follow Hendren (2013) so as not to exclude any purchasers of LTCI at the upper end of the age distribution.
